# Compliance with the European Pregnancy Prevention Programme in Isotretinoin Treatment: Safety Outcomes and Dose-Related Correlations

**DOI:** 10.3390/jcm14103497

**Published:** 2025-05-16

**Authors:** Piotr Brzeziński, Igor Jarosław Feszak, Janusz Śmigielski, Piotr Kawczak, Tomasz Bączek

**Affiliations:** 1 Institute of Health Sciences, Pomeranian University in Słupsk, 76-200 Słupsk, Poland; brzezoo77@yahoo.com (P.B.); igorfeszak@gmail.com (I.J.F.); 2 Department of Dermatology, Voivodeship Specialist Hospital, 76-200 Słupsk, Poland; 3 State School of Higher Professional Education in Konin, 62-510 Konin, Poland; janusz.smigielski@konin.edu.pl; 4 Department of Pharmaceutical Chemistry, Faculty of Pharmacy, Medical University of Gdańsk, 80-416 Gdańsk, Poland; tomasz.baczek@gumed.edu.pl; 5 Department of Nursing and Medical Rescue, Institute of Health Sciences, Pomeranian University in Słupsk, 76-200 Słupsk, Poland

**Keywords:** isotretinoin, pregnancy prevention programme, side effects, teratogenicity

## Abstract

**Background:** Isotretinoin is a highly effective treatment for moderate-to-severe acne, but strict contraceptive measures are required because of its teratogenicity. The European Pregnancy Prevention Programme (PPP) aims to minimise foetal exposure through structured protocols. However, real-world data on patient compliance and treatment outcomes are limited. **Methods:** This retrospective study included 569 female patients aged 14–25 years treated with isotretinoin in Poland (2021–2022). Patients were assigned to three groups based on PPP compliance: full (Group I), partial (Group IIA), and minimal (Group IIB). Data on contraception, cumulative dose, treatment duration, adverse events, laboratory monitoring, and therapy discontinuation were analysed using non-parametric statistical tests (*p* < 0.05). **Results:** No pregnancies occurred during treatment. Overall compliance with PPP requirements was high: 100% of the patients used contraception or declared abstinence. The majority (92.79%) used condoms, 1.93% used oral contraceptives, and 7.21% reported abstinence. Significant differences in cumulative isotretinoin dose were observed between the groups (Kruskal–Wallis H = 19.89, *p* < 0.001), with Group I receiving a lower mean dose than those in Groups IIA (*p* < 0.001) and IIB (*p* < 0.01). Notably, all therapy discontinuations (4.75%) occurred in Group I (full compliance), which may reflect stricter monitoring and an earlier identification of adverse effects or patient concerns. These discontinuations were associated with lower cumulative dosing (Mann–Whitney Z = 7.81, *p* < 0.001) than that seen in the other groups. An inverse correlation between age and cumulative dose was also found (H = 13.09, *p* = 0.0004), with younger patients (≤17 years) more likely to reach therapeutic targets. **Conclusions:** Isotretinoin therapy under structured PPP protocols is safe and effective, with no pregnancies reported and excellent contraceptive compliance. Significant differences in dosing and adherence patterns highlight the need for flexible patient-centred approaches to optimise safety and outcomes.

## 1. Introduction

**Figure 1:**
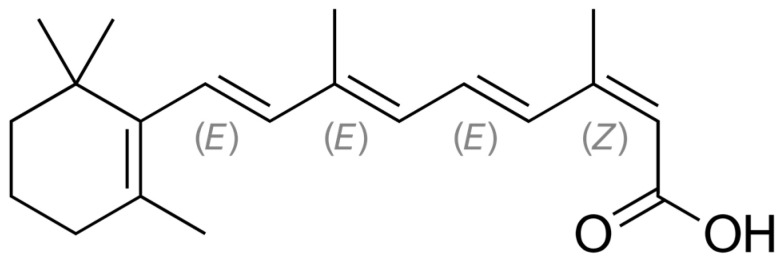
Structural formula of 13-cis-retinoic acid (isotretinoin) with the stereochemical configuration E–Z, an extension of cis–trans isomer notation, based on the study of [11].

Isotretinoin (13-cis-retinoic acid) is a synthetic vitamin A derivative classified as a first-generation retinoid (Figure 1). It was first synthesised in the 1950s and received approval from the United States (US) Food and Drug Administration (FDA) in 1982 for treating severe acne [1]. Structurally, isotretinoin is a stereoisomer of all-trans retinoic acid, and its biological activity is mediated through interactions with retinoic acid receptors (RAR-α, RAR-β, RAR-γ) and retinoid X receptors (RXR-α, RXR-β, RXR-γ) [2]. Through these interactions, isotretinoin influences the activity of nuclear receptors that regulate gene transcription, thereby affecting cell proliferation, differentiation, and apoptosis [3,4]. Isotretinoin is primarily prescribed for moderate-to-severe acne vulgaris, particularly in cases resistant to conventional treatments, including antibiotics and hormonal therapies. It is also effective for managing nodulocystic acne (acne conglobata), scarring acne, and adult acne [3,5]. In addition to acne, isotretinoin has demonstrated therapeutic benefits for treating rosacea, pustular psoriasis, lichen planus, frontal fibrosing alopecia, actinic keratosis, granuloma annulare, and cutaneous T-cell lymphoma [3,6,7]. This drug exerts its effects via multiple pathways. It significantly reduces sebaceous gland size and activity, resulting in up to a 90% decrease in sebum production [3]. Additionally, it inhibits sebocyte proliferation, has anti-inflammatory properties by downregulating toll-like receptor 2 and T helper 17 cells, and reduces colonisation by Cutibacterium acnes [8]. Another crucial mechanism is the normalisation of follicular keratinisation, which prevents the formation of microcomedones—the primary lesions in acne pathogenesis [3]. The efficacy of isotretinoin is well established, with 85–90% of patients achieving complete remission when treated with standard doses of 0.5–1.0 mg/kg/day, up to a cumulative dose of 120–150 mg/kg [9,10].

Common adverse effects include xerosis (dry skin), cheilitis (inflammation of the lips), erythema, increased photosensitivity, scaling, pruritus, conjunctivitis, and nail brittleness [12,13]. Systemic side effects may involve hyperlipidemia, hepatotoxicity, musculoskeletal pain, and psychiatric symptoms such as depression and anxiety [14,15]. Among these adverse effects, teratogenicity is the most serious concern associated with isotretinoin use. The drug is classified as a severe teratogen, and exposure during pregnancy results in irreversible congenital conditions. Teratogenicity is linked to the overactivation of retinoic acid receptors and retinoid X receptors in the developing embryo, leading to disruptions in embryogenesis and the differentiation of neural, mesenchymal, and epithelial tissues. This results in serious malformations affecting the central nervous system, cardiovascular system, thymus, craniofacial structures, and limbs [16,17]. It is estimated that 20–35% of foetuses exposed to isotretinoin develop major birth defects, while an additional 30–60% may experience neurocognitive impairment and developmental delays despite the absence of visible physical anomalies [16]. The most frequently reported abnormalities are microcephaly, hydrocephalus, microphthalmia, cleft palate, ear malformations, the tetralogy of Fallot, thymic agenesis, and limb deformities [16,18].

To prevent foetal harm, strict pregnancy prevention measures are in place in many countries. To reduce teratogenic risk from isotretinoin, women of childbearing potential must use at least two effective contraceptive methods (e.g., oral hormonal and barrier methods) for one month before, during, and one month after treatment. This is supported by mandatory pregnancy testing and consent programmes like iPLEDGE in the US [19,20,21] and the Pregnancy Prevention Programme (PPP) in the EU [22,23,24].

Despite growing awareness of these risks and available measures, patient compliance remains inconsistent across studies [24,25,26]. Therefore, we conducted a study to assess adherence in Poland.

## 2. Materials and Methods

### 2.1. Study Population and Design

We conducted a retrospective study of a randomised cohort of female dermatology patients in Poland. All participants were enrolled in the PPP before initiating isotretinoin therapy. Each patient received detailed counselling regarding teratogenic risks associated with isotretinoin use, including written and verbal information. A pregnancy test was performed within 3 days prior to the start of treatment (in accordance with the recommendation (PPP (EU)). When fertilisation occurs, the syncytial trophoblast secretes human chorionic gonadotropin (β-hCG), which can be detected in maternal serum approximately 6–8 days after fertilisation [27]. Serum β-hCG levels can confirm early implantation. This is followed by a rapid rise in median β-hCG levels that occurs early in pregnancy [28]. There is, of course, a risk of false negative pregnancy test results, and recent pregnancies may go undetected. On the other hand, performing pregnancy tests in individuals with certain medical conditions or in individuals taking medications containing hCG before testing may confirm the presence of β-hCG. The negative pregnancy tests in these groups may not fully exclude early, undetectable pregnancies. The patients were instructed to use one highly effective contraceptive method or two complementary methods simultaneously. Contraceptive use was mandatory, starting at least 1 month prior to treatment initiation and continuing throughout the treatment period. Only patients without contraindications for isotretinoin therapy were included. The first patient began treatment in July 2021 and completed it in May 2022. The last patient started therapy in November 2021, completed it in September 2022, and was followed up until October 2022. This study was conducted at two dermatological centres: the Department of Dermatology, 6th Military Support Unit in Ustka, Poland and the Department of Dermatology, Voivodeship Hospital in Słupsk, Poland. Oral isotretinoin was prescribed in weight- and severity-adjusted doses, considering the extent of acne lesions and the patient’s age. The study population consisted of females aged 14 to 25 years diagnosed with chronic moderate-to-severe acne. The patients were treated with one of the following daily doses: 20 mg, 123 patients (21.62%); 30 mg, 340 patients (59.75%); and 40 mg, 106 patients (18.63%).

### 2.2. Classification by European Pregnancy Prevention Programme Adherence

**Figure 2:**
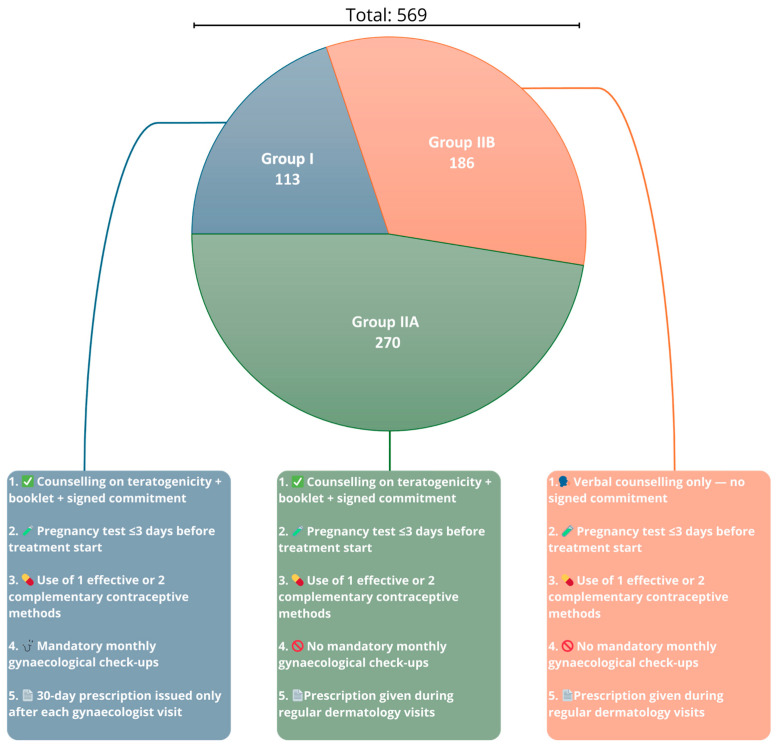
Distribution of patient subgroups and levels of Pregnancy Prevention Programme (PPP) implementation across Groups I, IIA, and IIB. Group I—patients under full PPP (Pregnancy Prevention Programme) compliance, including gynaecological visits and signed informed consent; Group IIA—patients with partial PPP compliance (without monthly gynaecological visits); and Group IIB—patients with minimal PPP compliance (verbal counselling only, without monthly gynaecological visits).

Women in Group I (Figure 2) strictly adhered to the PPP, fulfilling all mandatory elements and recommended precautions. (1) Each patient received comprehensive counselling regarding isotretinoin use and its teratogenic risks. They received an educational booklet and signed an informed consent form stating that they understood the potential effects of isotretinoin on the foetus. (2) A pregnancy test was performed within 3 days prior to treatment initiation. (3) The patients implemented one highly effective contraceptive method or two complementary methods. (4) They underwent mandatory monthly gynaecological consultations to verify compliance with contraception requirements. (5) Only after each gynaecological visit were they issued a prescription for isotretinoin, with the quantity limited to a 30-day supply. This group followed a stringent version of the PPP protocol to ensure maximum compliance and close monitoring throughout the treatment. Group II was divided into two subgroups (Figure 2), Group IIA and Group IIB, based on the level of adherence to the PPP components. Group IIA patients fulfilled the same requirements as those of Group I patients in the following aspects: (1) They received comprehensive counselling regarding isotretinoin use and its teratogenic risks, were provided with an educational booklet, and signed an informed consent form stating they understood the potential effects of isotretinoin on the foetus. (2) A pregnancy test was performed within 3 days prior to treatment initiation. (3) The patients implemented one highly effective contraceptive method or two complementary methods. However, unlike those in Group I, patients in Group IIA (4) were not required to attend monthly gynaecological check-ups. (5) Patients received isotretinoin prescriptions during regular monthly dermatological visits. Group IIB differed from Group I in the following points: (1) Patients received only verbal counselling about the side effects and teratogenicity of isotretinoin. They did not sign any formal commitment or informed consent form acknowledging the risks. (2) A pregnancy test was performed within 3 days prior to treatment initiation. (3) The patients implemented one highly effective contraceptive method or two complementary methods. (4) Similar to Group IIA, they were not required to undergo monthly gynaecological check-ups or pregnancy testing during treatment. (5) The prescriptions were issued during regular monthly dermatological consultations. This classification allowed for the comparison of compliance levels and the effectiveness of different implementations of the PPP protocol in preventing potential pregnancy during isotretinoin therapy.

### 2.3. Treatment, Monitoring, and Statistical Analysis

Treatment consisted of a fixed daily dose of isotretinoin (20, 30, or 40 mg), with the treatment duration tailored to the patient’s body weight, aiming for a cumulative dose of 120 mg/kg—a regimen supported by prior evidence as optimal for minimising relapse risk [9]. Before therapy, baseline laboratory assessments included liver function tests (alanine aminotransferase and aspartate aminotransferase), lipid profile (total cholesterol, low-density lipoprotein, high-density lipoprotein, and triglycerides), and serum transaminase levels. These tests were repeated after 2 months of treatment. If no clinically significant abnormalities were observed, further laboratory monitoring was scheduled every 6 months, as recommended by international guidelines [21].

Only patients managed according to routine clinical standards and national isotretinoin prescription protocols were included. No experimental procedures were introduced at any stage of data collection or patient care. To ensure patient confidentiality, all data were anonymised before analysis. This study adhered to the principles of the Declaration of Helsinki and the EU’s General Data Protection Regulation. All statistical analyses were conducted using Statistica software version 13.3. The distribution of continuous variables was assessed using the Shapiro–Wilk test. Because most variables did not follow a normal distribution, non-parametric tests were employed. The Mann–Whitney U test was used to compare differences between two independent groups, whereas the Kruskal–Wallis test was applied for comparisons among three or more groups. A *p*-value of <0.05 was considered statistically significant. This analytical framework enabled a robust evaluation of differences in PPP adherence and biochemical monitoring practices across various compliance subgroups, facilitating insight into the effectiveness of isotretinoin risk-mitigation strategies in real-world clinical settings.

## 3. Results

### 3.1. Baseline Characteristics of the Study Group

The average patient age was 23.78 years (±2.90). Of the participants, 189 (33.21%) were under 16 years of age, and 380 (66.79%) were 17 or older. Age did not appear to influence the acne clearance rate. The most frequently affected area was the face, which was affected in all 569 patients (100%), followed by the back, which was affected in 412 patients (72.41%). The location of the lesions did not affect the acne clearance rate. On average, treatment lasted 8.81 months (standard deviation [SD] 1.47), with the longest treatment lasting 12 months and the shortest 2 months. The patients’ weights varied from 35 kg to 105 kg, with a mean of 59.89 kg (SD 12.12). The most common side effect was mild cheilitis, which was observed in all 569 patients (100%), followed by retinoid dermatitis in 109 patients (19.16%), facial erythema in 33 patients (5.90%), nosebleeds in 23 patients (4.04%), skin itching in 11 patients (1.93%), muscle pain in 10 patients (1.75%), hair loss in seven patients (1.23%), mood changes in three patients (0.53%), and joint pain in two patients (0.35%). Slight increases in liver enzymes and serum lipids were noted in five patients; none of these patients discontinued treatment.

### 3.2. Statistical Analysis of Treatment by Study Group

The analysis showed significant differences in total isotretinoin dose depending on treatment method (*p* < 0.0001). The mean dose in Group I was 115.89 ± 34.89 (median = 130.43), which was significantly lower than in Group IIA (133.74 ± 9.66, median = 135.21; *p* < 0.001) and Group IIB (134.10 ± 7.07, median = 134.10; *p* < 0.01). However, the difference between Group IIA and IIB was not statistically significant (*p* > 0.05) (Table 1).

The analysis showed significant differences (*p* < 0.0001) in total dose depending on the duration of therapy: the mean dose level of 57.30 ± 18.58 (median = 54.54) in Group I was significantly lower in women with uninterrupted therapy than in the average level of 134.29 ± 6.94 doses (median = 133.33) in women who discontinued therapy (Table 2).

Isotretinoin therapy was interrupted in 27 patients (4.75%). All interruptions occurred in Group I, which comprised 23.90% of all participants: one (0.88%) after 2 months, nine (7.08%) after 3 months, eight (7.08%) after 4 months, seven (6.19%) after 5 months, one (0.88%) after 6 months, and one (0.88%) after 7 months of treatment, despite existing lesions in the course of acne and failure to achieve a cumulative dose (120–150 mg/kg of body weight). None of the 569 patients became pregnant, with 11 (1.93%) reporting the use of oral contraceptives [two (1.77%) in Group I and nine (7.96%) in Group II]; 528 (92.79%) reporting the use of a condom during sex; and 41 (7.21%) [30 (6.58%) in Group I and 11 (9.73%) in Group II] reporting abstinence.

### 3.3. Age-Related Differences in Treatment Outcomes

**Figure 3:**
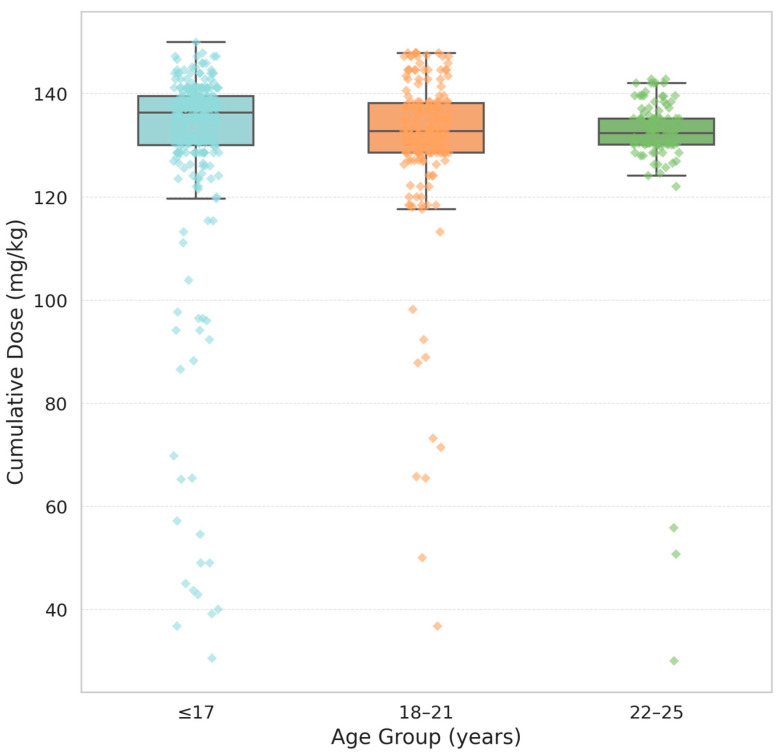
Cumulative dose by age group. Boxplots represent the distribution of cumulative isotretinoin doses (mg/kg) among three age groups: ≤17, 18–21, and 22–25 years. Each box illustrates the interquartile range (IQR), with the horizontal line indicating the median dose. The whiskers extend to the minimum and maximum values within 1.5 × IQR, while coloured diamonds denote individual outliers matching the colour of each group.

Because an inverse correlation was observed between patient age and cumulative isotretinoin dose, a non-parametric statistical analysis was applied to further explore this relationship. Patients were stratified into three predefined age categories: ≤17, 18–21, and 22–25 years. The cumulative dose, expressed as mg/kg of body weight, was the dependent variable. As the Shapiro–Wilk test revealed non-normal distributions within the groups, the Kruskal–Wallis test was used to assess differences in median dose values across age groups. The analysis yielded significant results (H = 13.09, *p* = 0.0004), indicating that younger patients (≤17 years) were more likely to complete therapy with a higher total dose. This trend was observed despite the absence of significant differences in the treatment duration or daily dosage based on age. This finding suggests that younger individuals are more motivated, more closely monitored by caregivers, or more compliant with follow-up requirements, allowing them to reach their therapeutic targets more effectively. Further studies are needed to determine whether psychosocial or adherence-related factors contribute to this pattern. These results are summarised in Table 3 and Figure 3, which show the cumulative isotretinoin doses stratified by age group.

## 4. Discussion

### 4.1. Overview of Risk Mitigation Systems: iPLEDGE and PPP

The iPLEDGE programme imposes strict regulatory measures to minimise foetal exposure during isotretinoin therapy. All patients, prescribers, pharmacies, and distributors involved in prescribing or handling isotretinoin must be enrolled in the system. Patients are required to acknowledge the drug’s teratogenic risks and adhere to a set of structured safety protocols [19,20,21]. Particularly stringent requirements apply to female patients of childbearing potential. These include the following: (1) a negative pregnancy test before treatment initiation, (2) monthly pregnancy testing during therapy, (3) the use of two simultaneous forms of contraception, or a declaration of complete abstinence, starting at least 30 days before treatment and continuing through therapy and for 30 days afterwards [19,20,21], and (4) monthly verification of compliance through the iPLEDGE system [19,20,21].

Despite these measures, iPLEDGE has not entirely prevented pregnancies during isotretinoin therapy. Between 2006 and 2017, more than 4600 pregnancies were reported, with a peak pregnancy rate of 0.65% during the programme’s first year [19]. In the following years, the annual rate stabilised between 0.3% and 0.5% [19]. Furthermore, comparative data suggest that iPLEDGE did not significantly reduce foetal exposure compared with that of the earlier SMART programme, raising questions about its long-term effectiveness and efficiency [19,20,21]. Growing support has emerged for refining the contraceptive requirements within the programme. The current iPLEDGE guidelines mandate the use of two forms of contraception, regardless of individual efficacy. However, Tier 1 contraceptive methods, such as intrauterine devices (IUDs) and subdermal implants, offer >99.5% effectiveness as standalone options, making additional methods potentially redundant in such cases [21]. In contrast, Tier 2 methods, such as combined oral contraceptives, have a higher failure rate when used alone (approximately 4.5% over 6 months), but their effectiveness increases to over 99% when combined with a secondary barrier method [21].

Table 4 summarises the specific requirements of patients receiving isotretinoin under iPLEDGE.

To mitigate foetal exposure to oral retinoids within the EU, the European Medicines Agency revised the PPP in 2018 to unify and strengthen risk minimisation measures across EU countries [22]. The updated PPP set detailed responsibilities for patients, prescribers, and pharmacists. Women of childbearing age must receive personalised counselling on teratogenic risks, sign a formal risk acknowledgement form, and receive a patient reminder card [22]. Prior to initiating therapy, a negative pregnancy test must be documented, with continued testing recommended monthly during treatment and once after discontinuation [22]. Contraceptive use is mandatory, with patients expected to use at least one highly effective (preferably user-independent) method—such as an IUD or implant—or two complementary methods (e.g., oral contraceptives plus a barrier method) [22]. Prescriptions are limited to a 30-day supply and can only be renewed if pregnancy testing and checklist verification have been completed [22]. Pharmacists and prescribers are responsible for confirming compliance; in cases of pregnancy during treatment, immediate referral to a teratology specialist is required [22].

Despite the comprehensiveness of this framework (see Table 2), evidence from multiple countries indicates that the implementation in clinical settings remains inconsistent. A large multi-country study (Denmark, Italy, the Netherlands, and Spain) involving over 88,000 retinoid users found no statistically significant reduction in retinoid use among women of childbearing age after the 2018 revision. Notably, pregnancy testing data were largely unavailable, and the reasons for treatment discontinuation were unknown in over 95% of the cases [22]. Contraceptive coverage showed a significant increasing trend in Spain alone. In other countries, the data were too limited to reliably assess this metric [22]. A study in Estonia involving 2575 women treated with isotretinoin between 2017 and 2020 found that 64.7% had no record of effective contraception despite PPP recommendations. Only 20.6% had complete contraceptive coverage for the entire risk window, and 17 pregnancies were identified during or shortly after isotretinoin treatment, translating to a risk of 6.6 per 1000 treated women [23]. The highest risk was observed in the 20–29 age group (11.6 per 1000) [23]. France exhibits a similar pattern. In a cohort of 272,723 women tracked between 2014 and 2021, compliance with monthly prescription renewals was high (98%), and most prescriptions were initiated by dermatologists (92.3%) [24]. However, pregnancy test compliance was low: 61% at treatment initiation, 72% during renewal, and only 25% at the end of treatment [24]. A public health campaign conducted in 2019 aimed to improve compliance; however, paradoxically, pregnancy test rates declined slightly upon renewal after its implementation [24]. Additionally, predictors of non-compliance include initiation by non-specialists, lower socioeconomic status, and initiation during the summer months [24]. These findings consistently underscore the disconnect between the regulatory guidelines and real-world practices. While the PPP outlines clear procedural safeguards, its successful implementation remains hindered by documentation gaps, system inefficiencies, and variations in healthcare access across populations.

Table 5 summarises the specific requirements for patients receiving isotretinoin under the PPP.

The iPLEDGE and PPP programmes are similar in purpose and structure; however, they have a few important differences (Table 6).

### 4.2. Interpreting Partial PPP Compliance

Although no pregnancies were observed in Groups IIA and IIB, this result must be interpreted with caution. These subgroups did not meet full PPP compliance, as they lacked critical elements such as monthly gynaecological monitoring and, in Group IIB, written informed consent. The absence of pregnancies may reflect chance, a limited sample size, or undetected early pregnancies due to the constraints of one-time pre-treatment testing. Therefore, these findings should not be viewed as evidence that partial compliance is equally effective or safe. According to current European guidelines, full adherence to PPP protocols—including dual contraception, structured monitoring, and formalised counselling—remains the only validated strategy for reducing isotretinoin-related teratogenic risk [22,23,24].

### 4.3. Contraceptive Strategies

Based on the article by Barbieri et al., contraceptive methods can be stratified into three tiers according to their effectiveness in preventing pregnancy, particularly during isotretinoin therapy. Tier 1 contraceptive methods—such as subdermal hormonal implants, IUDs (hormonal and non-hormonal), and permanent surgical contraception—demonstrate the highest efficacy, exceeding 99.5% during typical use over 6 months [21]. These methods are highly reliable as monotherapy and provide substantial protection against unintended pregnancy without the need for a secondary method. In contrast, Tier 2 methods, including depot medroxyprogesterone acetate injections and combined hormonal contraceptives (pill, patch, or ring), exhibit slightly lower effectiveness when used alone but can achieve >99% efficacy when paired with a secondary method, such as barrier protection [21]. Tier 3 methods, including barrier and fertility awareness-based methods, show significantly lower efficacy when used independently and are not recommended as primary contraception under iPLEDGE [21]. Recognising the differential efficacy of these tiers supports a more tailored and potentially less burdensome approach to contraception within the iPLEDGE framework.

**Figure 4:**
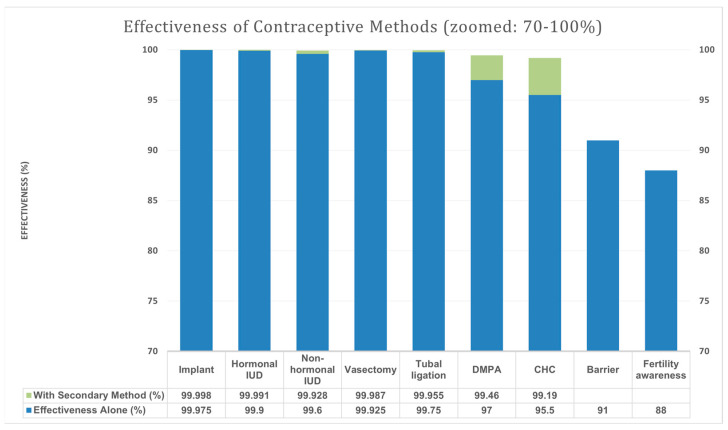
Effectiveness of contraceptive methods by tier based on the study of [21]. Blue bar—effectiveness (%) of the method alone. Green bar—effectiveness (%) with the addition of the secondary method. Secondary methods: male and female barrier methods, spermicidal agents, diaphragms, cervical caps, and vaginal contraceptive sponges; IUD—intrauterine device (a small contraceptive device inserted into the uterus); DMPA—depot medroxyprogesterone acetate (a long-acting injectable hormonal contraceptive); and CHC—combined hormonal contraceptives.

Figure 4 summarises contraceptive methods and their estimated effectiveness during 6 months of typical use.

Our study offers key insights into isotretinoin safety and treatment monitoring with a focus on contraceptive compliance among women of reproductive age. Notably, no pregnancies were recorded among the 569 participants despite the relatively low usage of Tier 1 contraceptive methods. Although our cohort primarily relied on condoms (Tier 3, 92.79%) or abstinence (7.21%), complete pregnancy prevention was achieved. This suggests that our safety protocols were clinically effective, even if statistical significance for contraception type and pregnancy outcome was not applicable.

This contrasts with several studies reporting suboptimal adherence and pregnancy-exposed cases. For instance, Ivask et al. found that 64.7% of isotretinoin-treated women in Estonia between 2017 and 2020 lacked dual contraception, resulting in a pregnancy exposure rate of 11.6/1000. Although their data demonstrated a significantly elevated risk (*p* < 0.01) among 20–29-year-olds [23], our cohort—with no pregnancies—highlights comparatively better outcomes.

In France, Havet et al. reported that only 25% of patients fulfilled mandatory pregnancy testing upon treatment completion, suggesting severe PPP implementation failures [24]. Their outcome lacked detailed statistical reporting on pregnancy incidence but showed a strong compliance gap.

When considering contraceptive method effectiveness, Barbieri et al. demonstrated through US-based modelling that relying solely on Tier 3 contraception (e.g., condoms) carries a 9% annual pregnancy risk versus < 0.5% for Tier 1 contraception [21]. Although our participants predominantly used the Tier 3 methods, no pregnancies occurred, which cannot be assumed to reflect statistical superiority without a comparator group.

Despite the proven effectiveness of Tier 1 and Tier 2 methods, 92.79% of our participants relied solely on condoms (Tier 3), while only 1.93% used hormonal contraception and 7.21% declared abstinence. This low uptake may reflect limited access, cost concerns, lack of awareness, or personal reluctance to use more effective methods. Improving education and access to long-acting reversible contraceptives (LARCs) may help address this gap. Another limitation is our reliance on self-reported contraceptive use, particularly in Groups IIA and IIB, where adherence was not routinely verified. Although no pregnancies occurred, this limits certainty. Group I had stricter monitoring protocols, including regular testing. Future studies should consider objective data sources, such as pharmacy records, to improve accuracy in measuring compliance.

### 4.4. Patient Age and Cumulative Dose: Statistical Correlation

A significant result of our study was the inverse correlation between patient age and cumulative isotretinoin dose (H = 13.09, *p* = 0.0004). This implies that younger patients were more likely to receive higher total dosing. Zane et al. also observed better treatment adherence among adolescents in the US, although their analysis did not report *p*-values for age-based dose differences [25]. Our findings were supported by a robust non-parametric Kruskal–Wallis test, confirming that age is a statistically significant predictor of treatment intensity.

Despite similar daily dosages and treatment durations across age groups, the cumulative doses differed, suggesting that younger patients may be more compliant during follow-up or less likely to miss doses. This aligns with Rademaker M et al.’s findings that failure to reach cumulative doses >120 mg/kg was strongly associated with relapse risk [16]. Likewise, James Q. Del Rosso noted that achieving a cumulative dose of 120–150 mg/kg is associated with longer periods of remission and a reduced risk of acne relapse [29].

### 4.5. Treatment Discontinuation

Therapy discontinuation in our cohort was low (4.75%) and limited exclusively to patients in Group I. Although this group had the strictest PPP adherence, it paradoxically demonstrated the highest dropout rate. This pattern was significant regarding treatment interruption timing and reduced cumulative dosing (*p* < 0.001). In contrast, Brzeziński et al. reported a much higher discontinuation rate (25.16%) in their Poland–Romania study, with psychiatric side effects as the leading cause [30]. Their data revealed a statistically significant correlation between psychiatric symptoms and early withdrawal. Similarly, Jakobi et al. found that 49.1% of previously treated individuals reported treatment concerns—particularly about depression—as negatively affecting adherence. Additionally, concerns were frequently influenced by non-medical sources, such as family and acquaintances, and included fears about mucocutaneous dryness, teratogenicity, and other somatic effects [31]. Hebebrand et al. reported that early mood-related symptoms often led to treatment discontinuation or dose reduction, even in the absence of confirmed psychiatric diagnoses. The lack of early clinical improvement and absence of family history of acne were also associated with a higher likelihood of dropout [32].

### 4.6. Biochemical Monitoring and Laboratory Findings

The biochemical findings of our study support previous research. Although we did not focus on laboratory monitoring, five patients showed elevated liver enzymes or lipids. This was similar to the results of Brzeziński et al., who found mild abnormalities in 4.82% of patients, which normalised post-treatment without clinical consequences [30]. These outcomes align with established recommendations for periodic biochemical screening, such as those from the British Association of Dermatologists [20].

Our findings add to the discussion on improving PPP adherence. In France, Raguideau et al. reported that PPP implementation was insufficient in over 50% of patients, increasing foetal risk [33]. By contrast, our study showed 100% real-world pregnancy prevention, despite some inconsistencies in testing. However, further research is needed to determine whether this reflects best practice or an isolated outcome. In addition to clinical outcomes, our findings contribute to ongoing conversations regarding laboratory surveillance during isotretinoin therapy [25,34,35]. While not a central focus of this study, we observed mild increases in liver enzymes and lipids in five patients (0.88%), none of which resulted in therapy discontinuation. These results are consistent with those of On et al., who documented laboratory abnormalities in 22% of patients receiving isotretinoin, most commonly elevated triglycerides and hepatic transaminases [36]. Werner et al. reported that 30% of patients required dose adjustments owing to laboratory deviations [37], highlighting the importance of biochemical vigilance.

Several important clinical studies have emphasised that laboratory abnormalities are rare and usually do not affect treatment. However, frequent laboratory monitoring is common practice. There is no need for extensive laboratory testing, but the importance of basic lipid and liver function tests is emphasised [38,39].

### 4.7. Patient Reporting and Behavioural Discrepancies

Another critical yet underappreciated challenge is the gap between self-reported and actual sexual behaviours. Tan et al. found that some women registered as abstinent in iPLEDGE later reported being sexually active, with 31% of sexually active women failing to use the two forms of contraception [40]. Although not formally tested for significance, this discrepancy implies risk underestimation in adherence metrics and supports the case for independent verification systems.

Digital health interventions and pharmacist-led strategies can help bridge these gaps. Havet et al. proposed integrating reminders and teleconsultations to increase PPP compliance [24]. Schaefer et al. noted that, of 18 live births among isotretinoin-exposed pregnancies in Germany, only one showed congenital anomalies [41]. This suggests that early detection and termination prevent most adverse foetal outcomes—but at ethical and social costs. Improved prevention, rather than post hoc management, must remain an objective.

Lastly, studies such as that by Lelubre et al. encourage greater access to Tier 1 contraceptives (e.g., implants and IUDs), possibly subsidised during isotretinoin treatment, especially in resource-limited settings [42]. Their policy-based approach emphasised long-acting reversible contraception as the most effective tool to reduce isotretinoin-related foetal exposure.

### 4.8. Limitations of Zero-Event Data Interpretation

Based on a previous paper that introduces a bivariate Poisson distribution via conditional specification, the key statistical insight is that a lack of observed events (e.g., pregnancies) does not necessarily indicate perfect effectiveness due to potential negative correlation and overdispersion in the count data [43]. The analysis showed that zero observed events can occur even when the underlying risk is not zero. Observed outcomes may be influenced by various contextual factors—for example, how the information is communicated to the patient and the pregnancy monitoring system used by the specialist initiating the treatment.

## 5. Conclusions

Isotretinoin remains one of the most effective systemic therapies for moderate-to-severe acne. Owing to its well-documented teratogenic potential, strict regulatory measures have been implemented in many countries, including the PPP in the EU and the iPLEDGE programme in the US. These frameworks emphasise patient education, the use of reliable contraception, and routine pregnancy testing throughout therapy. Despite the widespread adoption of these guidelines, real-world data on compliance and outcomes remain limited or inconclusive.

This study assessed adherence to PPP protocols and treatment outcomes in female patients undergoing isotretinoin therapy in Poland. The participants were divided into groups reflecting different levels of PPP implementation, from full adherence with gynaecological oversight to minimal application of the protocol. Remarkably, no pregnancies occurred during treatment, and all patients reported the use of contraception or abstinence, indicating full compliance with PPP requirements and supporting the clinical effectiveness of the prevention strategy.

Several important findings were observed. First, differences in cumulative isotretinoin dose emerged across compliance groups, with lower total doses seen in those with stricter protocol adherence. Second, treatment discontinuation was limited to the group with full compliance and was associated with lower cumulative dosing. These interruptions were not attributed to adverse effects but may have resulted from individual preferences or closer clinical oversight. Third, a trend was noted indicating that younger patients were more likely to complete treatment at optimal therapeutic levels. Mild elevations in liver enzymes or lipids occurred but did not lead to treatment cessation, supporting the safety of standard biochemical monitoring.

This study supports that isotretinoin therapy is safe and effective when conducted under structured protocols aligned with PPP principles. The absence of pregnancies and universal contraceptive use across all subgroups strongly supports the reliability and success of the implemented prevention strategy. Observed differences in dosing and adherence patterns offer valuable insights into optimising treatment and enhancing patient engagement in dermatological care.

## Figures and Tables

**Table 1:** Analysis of the total doses by therapy among all groups.

Parameters	Method of Treatment: Group I	Method of Treatment: Group IIA	Method of Treatment: Group IIB	Total
Number of respondents	113	270	186	569
Minimum	30.00	88.89	94.12	30.00
Maximum	147.94	147.94	150.00	150.00
Median	130.43	135.21	134.69	134.69
The arithmetic mean	115.89	133.74	134.10	130.31
Standard deviation	34.70	9.66	7.07	18.69
Asymmetry factor	−1.34	−2.24	−0.81	−3.39
Statistical analysis tests	ANOVA _Kruskal–Wallis_ H = 19.89 *p* < 0.001 (*p* = 0.000) Z^I,IIA^ _Mann–Whitney_ = −4.37 *p* > 0.001(*p* = 0.0000) Z^I,IIB^ _Mann–Whitney_ = −3.26 *p* < 0.01 (*p* = 0.0011) Z^IIA,IIB^ _Mann–Whitney_ = 1.33 *p* > 0.05 (*p* = 0.1824)			

Group I—patients under full PPP (Pregnancy Prevention Programme) compliance, including gynaecological visits and signed informed consent; Group IIA—patients with partial PPP compliance (without monthly gynaecological visits); Group IIB—patients with minimal PPP compliance (verbal counselling only, without monthly gynaecological visits); ANOVA _Kruskal–Wallis_ H—non-parametric test comparing the cumulative isotretinoin doses across the three study groups (Group I, Group IIA, and Group IIB); Z^I,IIA^—Z-score from Mann–Whitney U test comparing Group I vs. Group IIA; Z^I,IIB^—Z-score from Mann–Whitney U test comparing Group I vs. Group IIB; and Z^IIA,IIB^—Z-score from Mann–Whitney U test comparing Group IIA vs. Group IIB.

**Table 2:** Total dose analysis by therapy continuation or discontinuation.

Parameters	Discontinuation of Therapy: Yes	Discontinuation of Therapy: No	Total
Number of respondents	27	86	113
Minimum	30.00	118.40	30.00
Maximum	97.67	147.94	147.94
Median	54.54	133.33	130.43
The arithmetic mean	57.30	134.29	115.89
Standard deviation	18.58	6.94	34.70
Asymmetry factor	0.53	−0.10	−1.34
Statistical analysis test	Z _Mann–Whitney_ = 7.81 *p* < 0.001 (*p* = 0.0000)		

Z _Mann–Whitney_—Z-score derived from the Mann–Whitney U test comparing dose distributions between two independent groups (discontinued vs. completed therapy).

**Table 3:** Cumulative dose by age group.

Age Group	N	Mean Dose (mg/kg)	SD	Median	Min	Max
≤17	283	129.97	21.08	136.36	30.51	150.00
18–21	183	130.62	16.29	132.78	36.73	147.94
22–25	103	130.71	15.62	132.35	30.00	142.85

N—the number of patients (observations) included in a given age group; H—Kruskal–Wallis test statistic, used to assess whether there are statistically significant differences in cumulative isotretinoin doses between independent age groups (≤17, 18–21, and 22–25 years); a higher H value indicates greater divergence between group medians. In this analysis, H = 13.09, *p* = 0.0004, suggesting a statistically significant difference in dose distribution among the compared age groups.

**Table 4:** Summary of key requirements for participation in the iPLEDGE programme based on the studies of [19,20,21].

Who the Programme Applies to:	Requirements:
All Patients	Must register in the iPLEDGE system Must provide informed consent Must understand the teratogenic risks of isotretinoin
Female Patients of Childbearing Potential (FCBP)	Negative pregnancy test before starting treatment Monthly negative pregnancy tests during treatment Use of two forms of contraception or strict abstinence Must begin contraception 30 days before starting treatment and continue throughout and 30 days after therapy Monthly verification of compliance via iPLEDGE system
Patients Declaring Abstinence	Must confirm abstinence monthly Must acknowledge that approximately 20% of patients reporting abstinence are sexually active during treatment
Male Patients	Must register in iPLEDGE Must be informed of the teratogenic risks (even though foetal risk from male exposure is negligible)
Prescribers (Physicians)	Must be registered and certified in iPLEDGE Responsible for confirming pregnancy test results and counselling on contraception
Pharmacies	Can only dispense isotretinoin after confirming iPLEDGE compliance Prescription must be filled within 7 days of authorization for FCBP patients
Wholesalers/Distributors	May only distribute isotretinoin to registered and compliant pharmacies and prescribers

FCBP—female patients of childbearing potential; iPLEDGE—isotretinoin pregnancy linked evaluation and distribution system; All Patients—all individuals prescribed isotretinoin regardless of sex or reproductive potential; Prescribers—physicians authorised to prescribe isotretinoin; Pharmacies—licenced facilities dispensing isotretinoin; and Wholesalers/Distributors—entities supplying isotretinoin to pharmacies and prescribers.

**Table 5:** Summary of key requirements in the European Pregnancy Prevention Programme (PPP) for oral retinoids based on the studies of [22,23,24].

Who the Programme Applies to:	Requirements:
All Patients	Must receive counselling on the teratogenic risks of isotretinoin Must sign a risk acknowledgment form Must receive printed educational materials (e.g., reminder card)
Female Patients of Childbearing Potential (FCBP)	Must take a pregnancy test before starting treatment (ideally within 3 days) Should undergo monthly pregnancy tests during treatment Must take a pregnancy test after stopping treatment Must use either one highly effective contraceptive method or two complementary methods Contraception must start ≥1 month before, continue during, and for ≥1 month after treatment
Patients Declaring Abstinence	Must confirm abstinence during counselling Still required to follow PPP requirements, including pregnancy testing
Male Patients	No specific contraception or testing requirements Must be informed of teratogenic risks and avoid sharing medication
Prescribers (Physicians)	Must provide counselling and confirm understanding Must complete and retain a prescriber checklist Must verify pregnancy tests and contraception before prescribing Must monitor compliance throughout treatment
Pharmacies	Must confirm checklist completion before dispensing May dispense only a 30-day supply Early refills are not allowed
Wholesalers/Distributors	No formal role in PPP system Expected to supply isotretinoin only to authorised facilities

PPP—Pregnancy Prevention Programme; FCBP—female patients of childbearing potential; IUD—intrauterine device; All Patients—all individuals prescribed isotretinoin regardless of sex or reproductive potential; Prescribers—physicians authorised to prescribe isotretinoin; Pharmacies—licenced facilities dispensing isotretinoin; and Wholesalers/Distributors—entities supplying isotretinoin to pharmacies and prescribers.

**Table 6:** Comparison of key requirements: iPLEDGE (United States) vs. Pregnancy Prevention Programme (European Union) based on the studies of [19,20,21,22,23,24].

Category:	iPLEDGE (USA):	PPP (EU):
Programme Type	REMS, mandated by FDA	RMM, mandated by EMA
Patient Enrollment	Mandatory central registration	No central system; local documentation
Patient Education	Counselling + signed form + printed materials	Same as iPLEDGE
Pregnancy Test (PRE)	Required within 7 days before prescribing	Ideally within 3 days before treatment
Pregnancy Test (Ongoing)	Required monthly, logged in system	Recommended monthly
Pregnancy Test (Post)	Not required, but contraception must continue 1 month after	Required after treatment to confirm no pregnancy
Contraception	Two methods of abstinence	One effective or two complementary methods
Contraception Timing	Start ≥1 month before, during, and 1 month after	Same as iPLEDGE
Prescription Limit	30-day supply; expires in 7 days	30-day limit; renewal only with updated documentation
Prescriber Duties	Monthly documentation in system; verify all requirements	Complete checklist; ensure all measures are met
Pharmacist Duties	Confirm compliance in iPLEDGE; electronic authorization	Verify checklist; dispense max 30 days
If Pregnancy Occurs	Stop treatment; refer to specialist	Same as iPLEDGE
Gender Policy (Post-2021)	Based on reproductive potential (gender-neutral)	Based on biological sex; no EU-wide gender-neutral approach
Digital Burden/Access	Online system often cited as barrier	Less centralised; variable implementation

iPLEDGE—isotretinoin pregnancy linked evaluation and distribution system; PPP—Pregnancy Prevention Programme; REMS—risk evaluation and mitigation strategy; RMM—risk minimization measure; FDA—Food and Drug Administration; and EMA—European Medicines Agency.

## Data Availability

The data presented in this study were collected and recorded in a manner that ensures anonymity. Data are available upon request from the corresponding author. The data are not publicly available due to privacy and ethical restrictions.
